# Dairy Matrix Effects: Physicochemical Properties Underlying a Multifaceted Paradigm

**DOI:** 10.3390/nu16070943

**Published:** 2024-03-25

**Authors:** Thom Huppertz, Blerina Shkembi, Lea Brader, Jan Geurts

**Affiliations:** 1 Food Quality & Design Group, Wageningen University & Research, 6808 WG Wageningen, The Netherlands; 2 FrieslandCampina, 3818 LE Amersfoort, The Netherlands; jan.geurts@frieslandcampina.com; 3 Arla Innovation Center, 8200 Aarhus, Denmark

**Keywords:** product matrix, matrix effect, dairy, nutrition, health, digestion, absorption, product structure, component interactions

## Abstract

When food products are often considered only as a source of individual nutrients or a collection of nutrients, this overlooks the importance of interactions between nutrients, but also interactions between nutrients and other constituents of food, i.e., the product matrix. This product matrix, which can be defined as ‘*The components of the product, their interactions, their structural organization within the product and the resultant physicochemical properties of the product*’, plays a critical role in determining important product properties, such as product stability, sensory properties and nutritional and health outcomes. Such matrix effects can be defined as ‘*the functional outcome of specific component(s) as part of a specific product matrix*’. In this article, dairy matrix effects are reviewed, with particular emphasis on the nutrition and health impact of dairy products. Such matrix effects are critical in explaining many effects of milk and dairy products on human nutrition and health that cannot be explained solely based on nutrient composition. Examples hereof include the low glycemic responses of milk and dairy products, the positive impact on dental health, the controlled amino acid absorption and the absence of CVD risk despite the presence of saturated fatty acids. Particularly, the changes occurring in the stomach, including, e.g., coagulation of casein micelles and creaming of aggregated fat globules, play a critical role in determining the kinetics of nutrient release and absorption.

## 1. Introduction

The consumption of nutritious and healthy food is essential for the maintenance of the health of mankind. In essence, the foods consumed by a person should ideally provide the daily requirements for all essential nutrients in a safe manner. Provision in a safe manner, of course, involves that foods are free of toxins or other contaminants, as well as free of microbiological hazards. However, it also demands that the nutrients be provided in such a way that the food does not contribute to non-communicable diseases. The main dietary risks in relation to non-communicable diseases have been outlined in the global burden of disease (GBD) studies and include dietary risk factors (DRFs) and diets containing either too much or too little of certain food groups or specific components [1]. Such effects typically go beyond nutrient sufficiency alone because, while consumption of, e.g., red meat or processed meat may make significant nutrient contributions [2,3], their consumption is also linked to increased risks in, e.g., cardiovascular diseases [1]. Likewise, while fruit makes a comparatively limited contribution to overall nutrient intake [4], diets low in fruit are considered one of the main dietary risk factors [1]. This creates a paradox because, while nutrient contents of foods are easily determined, they can only provide indications towards (potential) nutrient sufficiency (assuming sufficient bioavailability); however, considering products solely based on nutrient content and composition can only be applied to DRFs related to specific nutrients, e.g., those related to diets high in sodium or low in calcium. However, nutrient-based approaches do not consider any DRFs attributable to specific food groups, e.g., DRFs attributed to diets low in fruit, low in milk or high in red meat or processed meat.

Particularly in relation to the predicted rapidly evolving shifts in dietary patterns in relation to food production within planetary boundaries [5,6], the aforementioned discrepancy between information from nutrient contents alone and DRFs is important to consider. Current DRFs, as determined in the GBD studies, are based on a long history of epidemiological studies on food groups and food products that have long been part of the human diet. However, for new food products, epidemiological evidence is lacking and, given the type of research behind it, will not be available for many years to come.

As such, any potential assessment of the contributions of the products in nutritious and healthy diets can only be made on the basis of nutrient composition. This, however, entails significant uncertainty and risks, which is exemplified when considering health risks attributed to saturated fatty acids (SFAs). Much of the presumed evidence for health risks related to SFA consumption was based on isolated component studies, which were subsequently extrapolated by association to food products containing substantial levels of SFAs, e.g., dairy products or meat products, chocolate, biscuits or cakes or products in which, e.g., milk fat or palm fat were used as ingredients. Despite the fact that meta-analyses do not support these apparently oversimplistic extrapolations [7,8,9,10,11,12] and have been repeatedly challenged, generic recommendations on SFA intake have often remained and have not been considered on a product (group) basis. This, however, is critical, as holistic considerations of product matrices, and even meal matrices, cannot be ignored [13].

In this review, we discuss the topic of food matrix effects, with specific emphasis on dairy products. We will focus first on defining matrices and matrix effects both inside and outside of the scope of nutrition and health. Subsequently, examples of matrix effects of dairy products in relation to nutrition and health are discussed. In the final section, we will discuss a further frontier, i.e., looking beyond individual products and their matrices and considering interactions on a meal basis. The focus of this review is primarily on matrix effects related to milk and dairy products from bovine milk.

## 2. Matrices and Matrix Effects

Much is known about the individual constituents of milk and dairy products, and several milk constituents are isolated to highly purified forms (e.g., lactose, milk fat or milk protein preparations). However, these constituents are rarely consumed in isolation and are typically consumed in the form of multicomponent systems, i.e., food products, which can be described based on composition but also based on the structural organization of the components in the system. Both composition and product structure can notably affect the effect of a given nutrient, as has been demonstrated in the case of saturated fatty acids (SFAs). Based on previous studies, including meta-analyses, SFA intake has been associated with an increased risk of cardiovascular disease, resulting in dietary advice in several countries to limit the intake of SFA. However, more recent research has shown that the effects of SFA are dependent on the product via which they are consumed. For cheese, for instance, SFA content would be expected to increase CVD risk, but the opposite is found [14,15,16,17]. The same is seen for SFA consumption SFA intake from chocolate and beef [13]. These findings strongly highlight the importance of holistic product-based approaches rather than single-nutrient approaches and that findings from single-nutrient studies in one (model) system cannot be simply extrapolated to other products based solely on concentrations of that nutrient.

This dependence on the effects of a nutrient or a nutrient class on the product via which it is consumed is commonly referred to as the ‘matrix effect’ and has been a key topic of research and debate over the past decade. The term ‘matrix effect’ is unfortunately often ill-defined and, as such, runs the risk of becoming a panacea for any aspects not readily explainable. In chemical analysis, a ‘matrix’ is commonly defined as the components of a sample other than the analyte of interest, and the effects of these other components on the outcomes of the analysis are called matrix effects [18,19,20,21]. Indeed, this definition of ‘matrix effects’ is very appropriate and can be the basis for understanding why the effects of nutrients differ depending on the product in which they are consumed.

However, we feel that in addition to the compositional aspect, a structural component should also be considered because, for products of (virtually) the same composition, the physical form in which it is consumed can affect outcomes. This was already illustrated almost five decades ago [22] when it was shown that plasma glucose and serum insulin levels after consumption of apples differed markedly for whole apples, apple puree or apple juice. Product structure for lentils also affects blood glucose levels [23], whereas digestion and absorption of amino acids differ for minced beef versus beef steak [24].

In addition to the aforementioned effect of macroscopic product structure, effects of product structure at a (sub)microscopic level can also affect digestion and absorption of nutrients. This can be observed by the differences in the rate of digestion and amino acid uptake of proteins from milk heated to different degrees due to heat-induced whey protein denaturation and its effect on gastric coagulation and gastric transit [25].

Therefore, we believe that an accurate definition for the matrix of a food product should consider both a compositional and a structural aspect, i.e.,

*‘The product matrix refers to the components of the product, their interactions, their structural organization within the product and the resultant physicochemical properties of the product.’*

In this context, the matrix thus includes a compositional part (i.e., which components are present in the product), a structural part (i.e., how are the components organized) and a physicochemical part (i.e., what is the result of the components and their structural organization on the properties of the product). The physicochemical properties of the product include, e.g., pH, buffering capacity, rheological properties, particle size, etc. We specifically use the word ‘components’ rather than ‘nutrients’ because not all components of food products are classified as nutrients, but these components can still have a notable effect on the structure and physicochemical properties of the food products.

Any specific effect derived from the aforementioned product matrix is referred to as a ‘*matrix effect*’. Such effects may be related to physiological outcomes, which is the primary focus of this review, but can also be related to other areas, e.g., physical stability or sensory properties of products.

In terms of physical stability, milk is a perfect example of how product matrix effects ensure physical stability. The amounts of calcium and phosphate in bovine milk, for instance, far exceed the solubility of the sparingly soluble calcium phosphate salts [26]. In a simple solution, this would lead to the undesirable formation of a precipitate of calcium phosphate in the form of a sediment. In milk, however, the presence of both citrate, which forms soluble complexes with calcium, and caseins, which encapsulate calcium phosphate into the casein micelles, means that the bovine milk can readily provide approximately 10 times more calcium in stable and bioavailable form than would be possible in a simple aqueous solution [27,28,29].

The presence of milk fat in the form of milk fat globules is another example where the matrix, in this case the emulsification and stabilization of fat in emulsion droplets, provides physical stability that would not be found in simple mixtures of water and fat [30,31]. In terms of matrix effects and their relation to sensory properties, one can consider the specific effects of rheological properties of the matrix on sensory perception. For instance, the same concentration of sugar or salt will be perceived as less sweet or salty when consumed as a gelled product rather than as a liquid [32,33,34,35,36]. Furthermore, suppression or enhancement of sensory perception by specific combinations of components is, of course, also a key example of the product matrix showing specific effects due to the combination of components.

In general, a product matrix effect can thus be defined as:

*‘The functional outcome of specific component(s) as part of a specific product matrix’*

Within the context of this article, however, we will focus primarily on effects of the product matrix on physiological outcomes and therewith define a matrix effect in that context as:

*‘The physiological outcome of the intake of component(s) in the form of a specific product matrix’*

Such matrix effects thus include those arising from specific compositions, the structural organization of the components and the resulting physicochemical properties. Matrix effects can also be considered on a molecular level. For carbohydrates, digestibility, as an effect, is related to whether monosaccharides are linked via an α- or β-glycosidic bond, whereas for proteins, the sequence of amino acids will also determine its digestibility; for fats, digestibility is determined by the distribution of fatty acids over the glycerol backbone in triglycerides.

Matrix effects can be both positive and negative. Earlier examples of the link between SFA and CVD when consumed, e.g., in the form of cheese, beef or chocolate [13], are examples of positive matrix effects. However, the reduction in bioavailability of calcium due to the presence of oxalate and phytate in some food products is a negative matrix effect [37,38]. Likewise, the presence of protease inhibitors in some (mainly plant-based) foods is also negative, as it can impair protein digestion [39,40].

As is clear from the above examples, digestion and absorption of components play a key role in matrix effects related to physiological outcomes that have been reported. In this respect, it is important to consider both the degree of digestion and absorption as well as the kinetics of the processes. In addition to the intestinal phase, where most of the absorption occurs, the gastric phase of digestion also plays a key role via pre-digestion of lipids and proteins by gastric lipase and pepsin, respectively, but also by controlling the rate of transit of components to the intestine [41,42]. A prime example, in this case, is the coagulation of the caseins in milk in the stomach, leading to a gradual and sustained release of proteinaceous material to the intestine [43,44].

## 3. Product Matrix Effects: Examples of Dairy Foods

As outlined previously, we believe that the term ‘product matrix effect’ is applicable in a broader context than solely physiological effects and is, e.g., equally applicable in the areas of, e.g., product stability and sensory properties of products. Also, here, it is not single components that determine the overall stability of sensory perception of products, but it is also the interplay between the components, their structural organization and the resulting physicochemical properties that determine the final outcomes. However, when considering these three different areas, i.e., physiological effects, product stability and sensory properties, one can distinguish the different levels of evidence available and applied in the field to bridge the gap between the ‘matrix’ of a product and the ‘matrix effect’ attributed to the product through mechanistic understanding.

### 3.1. Product Matrix Effects in Relation to Product Stability: Milk and Cheese

When considering liquid milk as an example, the stability of milk has been the topic of more than a century of research, and the basic principles have been established based on physical, chemical and biochemical principles that are widely applicable. From a microbial perspective, the microbiota of raw milk is well-known, both in terms of pathogens and spoilage bacteria, as well as their conditions of growth and the conditions based on which they are inactivated [45,46]. This is, in essence, applied in strictly controlled supply chains in the dairy sector and facilitates the supply of safe and stable products to the consumer [47,48]. In addition to microbial stability, the physical stability of milk is another topic that can be well understood in the context of matrix effects. Physical instability of milk can, for instance, be found in the form of the creaming of fat globules [30], the formation of a proteinaceous sediment, or even the gelation of milk under certain conditions [49]. In all cases, mechanisms for (potential) instability can be understood and based hereon, remedies can be designed. For instance, the creaming of milk fat globules can be readily understood based on the density difference between the fat and the skim milk, the size of the fat globules, their tendency to aggregate and the comparatively low viscosity of milk [30]. Based hereon, corrective measures by reducing fat globule size (e.g., through homogenization) can be applied to increase the stability of creaming. Thus, with the same components but a different structural organization and different resulting physicochemical properties, a different physical stability can be achieved; in other words, a processing-induced change in the product matrix led to a different matrix effect in terms of physical stability. For milk gelation and sediment formation, primary causes have been identified, including, e.g., the role of endogenous and bacterial enzymes and acid production by bacteria, all of which can cause protein instability [49,50]. Also, here, understanding the underlying mechanisms allows for the identification and implementation of adequate control factors via milk quality, processing and control of storage conditions. While mineral precipitation is typically observed in many plant-based drinks that are fortified with sparingly-soluble calcium salts (e.g., calcium phosphate or calcium carbonate [51]), this does not occur in milk because calcium phosphate in milk is encapsulated in casein micelles [28,52]. These micelles thus provide the ability to transport high levels of calcium phosphate without the risk of precipitation. Overall, for milk, it is thus apparent that matrix effects relating to the physical stability of the product are well-understood based on an understanding of the physicochemical properties of the product, the effects of biochemical reactions and processing thereof; based hereon, appropriate measures can be taken to minimize the risk of instability.

Like milk, a similar depth of understanding of the physiochemical properties of the product and potential mechanisms of instability and their control is available for many cheese varieties. The microbial stability of cheese products can relate to the growth of pathogenic and spoilage bacteria and includes both the microbiota of the milk but also the properties of the cheese, which will affect the outgrowth of bacteria [53,54,55]. The microbiota of cheese and control thereof by processing is based on similar principles as described above for milk. For cheese properties affecting the outgrowth of microorganisms, much research has been undertaken to understand these factors. This includes, amongst others, the acidity of the cheese product [56], the salt content [57,58], the potential of some lactic acid bacteria to produce bacteriocins [59,60], and the antimicrobial activities of some peptides formed during the ripening of some cheese varieties [61,62,63]. This understanding allows the creation of a cheese matrix that is safe and stable through processing, composition and culture selection.

### 3.2. Product Matrix Effects in Relation to Sensory Properties: An Example of Cheese

As for stability, as discussed above, matrix effects are also important in determining the sensory properties of food products. However, although the stability of products can, to a large extent, be understood by physicochemical and biochemical relations in the product matrix and its interaction with the environment, sensory properties involve another important component, i.e., the interaction of the product matrix and its components with those parts of the human body that are involved in sensory perceptions and how stimuli resulting therefrom, combined with audio and visual stimuli, are transferred to the brain to evoke an overall sensory perception [64]. The overall sensory perception of food product matrices is thus the result of the combination of characteristics of the product matrix detectable by the human senses, i.e., odor, taste, texture, appearance and sound, and each of these aspects is again the combination of many individual smaller parts [65]. For instance, to any individual, the overall flavor of a food product depends on the types of flavor compounds present, the concentrations at which they are present, the extent to which the flavor compounds are released in the mouth and come into contact with receptors and the sensitivity thresholds of individuals to perceive these flavor compounds [65]. The individual’s interpretation of this flavor profile may be perceived as a positive or negative appreciation based on factors such as familiarity, learning, culture and psychology, which, combined with an appreciation of odor, texture, appearance and sound can give an overall appreciation of the product [66,67,68]. Overall, it is thus clear that while matrix effects are crucial for understanding and modulating sensory aspects of products, they have a much higher level of complexity than stability because, in addition to the physicochemical properties of the food product matrix, there is an additional complicating factor, which is perception and translation by the human body and mind. Although much progress has been made and still is being made in terms of understanding all areas of sensory perception by humans, it is equally apparent that completing this understanding will take decades or, more likely, centuries. Hence, the very strong theoretical basis that is available for matrix effects relating to food product stability is not yet available to the same extent for matrix effects relating to sensory production of food products. As a result, ‘dealing with matrix effects’ in relation to sensory properties of food products requires different approaches, often focused on sensory evaluation of food products by trained sensory panelists and linking their outcomes to instrumentally determined properties of the food product matrix. Using these approaches, many advances have been made, and some examples relating to cheese are outlined below.

When considering the sensory perception of cheese, odor, taste, texture and appearance play a crucial role; sound, on the other hand, does generally not play a dominant role in the sensory perception of cheese products. In terms of appearance, one can consider the appearance of the body of the cheese, with a large number of eyes in Emmental cheese, some small eyes in Gouda cheese and no eyes in Cheddar cheese, but also the color of the cheese, which may range from near-white (e.g., for Feta cheese) or yellow/orange for cheese variants to which coloring is added [69]. Texture can vary widely for cheese products, with cheeses classified from soft to extra-hard, and is determined by gross composition, pH, degree of protein breakdown, calcium content and various other factors [70,71,72,73]. The odor of cheese is determined by the type and concentration of volatile compounds present, as well as their partitioning between the product and the air [74,75,76,77,78], whereas the taste is determined by water-soluble compounds, such as peptides, free amino acids and other metabolites [79,80,81] but also the salt content of the cheese [57,82]. For many of these factors, individual pathways have, to a large extent, been characterized qualitatively and to some extent also quantitatively. However, complexity arises in the interrelationship between pathways and factors. One example is the role of starter bacteria in cheeses, which are added for various reasons, i.e., (1) to convert lactose into lactic acid and other metabolites, thereby reducing pH and increasing acidity, (2) to contribute to the formation of flavor and odor compounds via proteolysis, lipolysis and glycolysis pathways, (3) to contribute to texture development via proteolysis, (4) to contribute to eye formation via production of CO_2_ or C_3_H_6_O_2_ [83,84]. Therefore, starter cultures affect the flavor, odor, texture and also the appearance of the product. However, there is also a strong interaction between the different parameters affected by the starter cultures. For instance, eye formation in cheese is strongly dependent on cheese texture, with an elastic texture required [85,86]. Elasticity, in turn, is strongly dependent on factors such as cheese pH and protein breakdown [72,87]. Textural parameters in cheese, in turn, also strongly affect the release of flavor compounds and, therefore, sensory perception [88,89]. Hence, it is clear that many factors are interlinked, underpinning the complexity of matrix effects in relation to even the influence of a single factor, i.e., starter cultures, on the sensory properties of cheese.

Likewise, the coagulant used in cheese-making, part of which remains in the cheese, also affects flavor and texture directly via protein hydrolysis, leading to increased levels of peptides and weakening of the casein structure [72,90,91]. However, coagulant in the cheese matrix also affects appearance and odor. The effect on appearance can be seen, e.g., in, as outlined above, eye formation, which is dependent on cheese texture, which, in turn, is partly controlled by the degree of protein hydrolysis. In terms of odor, residual coagulant also impacts this, as the peptides released from the primary proteolysis by the coagulant are broken down further to peptides and free amino acids, which can form the precursors for the formation of volatile odor compounds by lactic acid bacteria.

From the above, it is clear that matrix effects are crucial when it comes to controlling the sensory properties of cheese. It is also apparent that there is no generic cheese matrix but that every cheese variety will have its own matrix, which is also subject to change during the ripening of cheese due to many ongoing biochemical reactions. A further complexity, as outlined above, is introduced by the fact that the sensory properties of a product are highly complex, involving both product properties, the human senses and the interpretation thereof via familiarity, learning, culture and psychology. Despite this complexity, the overall complexity is somewhat reduced due to the fact that sensory effects are relatively short term (i.e., seconds to minutes after consumption), in contrast to, e.g., effects of human health, which, as outlined in the next section, can span over days, weeks, months, years or even decades.

Due to the relatively short time-scale of testing required to evaluate the effects of changes in the product matrix on the sensory properties of cheese, as well as the non-invasiveness of the testing, it has been possible to identify key pathways affecting the sensory properties of cheese, including, e.g., the formation of key volatile and non-volatile compounds, their pathways for formation, and their combined effects on sensory perception, which also include texture and visual appearance [69,70,74,92,93]. This understanding allows tailoring of cheese products to specifically desired sensory properties via, e.g., composition, selection of coagulant and starter culture and ripening conditions, and has also allowed effective strategies to counter undesired changes in sensory properties of cheese, e.g., on reducing fat content or salt content [94,95,96]. Overall, it is clear that when considering the sensory properties of cheese, or any other dairy or food product for that matter, product matrix effects are crucial. Effects observed cannot be explained, understood or steered based on single components or their concentrations but require conceptualization of the product matrix and how even subtle changes herein can have a large overall effect.

### 3.3. Product Matrix Effects in Relation to Human Nutrition and Health

Product matrix effects relating to sensory properties (Section 3.2) have a much higher complexity than those relating to product stability (Section 3.1) because they include interactions within the product as well as between the product and the human body. These effects, however, are still on relatively short timescales, i.e., seconds or minutes. Product matrix effects in relation to human health, however, occur on much larger timescales, which can range from days or weeks to years or decades. This already makes the interpretation of product matrix effects in relation to health benefits much more complex than product stability or sensory properties.

Further complexity is added due to the fact that long-term effects on health outcomes often involve extremely complex pathways in the human body, some of which have not been unraveled yet. Associations between consumption of specific food products (or classes thereof) and health outcomes are typically based on epidemiological or intervention studies or meta-analyses based thereon. However, while the outcome of epidemiological studies can be a statistically valid association, they have no role in causal inference. Causality can be obtained from randomized control trials (RCTs), but these studies are typically not carried out at the timescales over which health outcomes manifest. RCTs, therefore, often focus on the short-term effects of markers that are (believed to be) linked to long-term health outcomes rather than on actual health outcomes. The (potential) invasiveness and cost involved in RCTs of course provide some limitations as well, and they cannot be implemented as widely as, e.g., sensory panels, to assess sensory properties.

Despite the higher level of complexity in linking the product matrix of foods to (markers for) health outcomes, it has become abundantly clear that also in the area of nutrition and health, single-component approaches do not suffice, and product matrix effects need to be considered [97,98,99]. In this respect, two aspects of product matrix effects may be distinguished, i.e.,

(1) RCTs or other mechanistic studies, sometimes complemented with in vitro studies, to demonstrate the occurrence of product matrix effects, often on intermediate markers in relation to health outcomes.
(2) Epidemiological studies demonstrating associations between consumption of certain products and health outcomes, wherein product matrix effects are put forward as potential explanations for observed associations.

## 4. Mechanistic Insights on Dairy Product Matrix Effects

### 4.1. Dairy Protein Digestion and Amino Acid Absorption

The relationship between dairy and muscle health has been widely investigated in relation to, e.g., the growth of infants and young children, sports nutrition and sarcopenia [100,101,102]. Much focus in this area has been dedicated to milk proteins, particularly bovine milk proteins, with key topics of amino acid composition and protein digestibility, which center around the fact that sufficient nitrogen and essential amino acids are required for the synthesis of muscle protein and sufficient leucine to trigger muscle protein synthesis in adults. However, it should be realized that other factors, such as calcium, phosphate and vitamin D, also play a key role in muscle function [103,104].

A large body of mechanistic research has been dedicated to the digestion of bovine milk proteins and the influence of the product matrix thereon. Central to this topic has been a series of RCTs focusing on aminoacidemia and muscle protein synthesis after consumption of milk proteins in different matrices, supplemented with in vitro studies for additional mechanistic insights. From such studies, it has become clear that both caseins and whey proteins are well digested but that the kinetics of the digestion process differs. Whey proteins typically result in quicker post-prandial aminoacidemia than caseins, which can be related to the fact that, in milk, the caseins, at least when in micellar form, are subject to gastric coagulation and are thus released in a more gradual manner to the small intestine for further hydrolysis and subsequent uptake into the bloodstream [25]. In milk, the combination of caseins and whey proteins has been suggested to provide a rapid peak in blood amino acids from the whey proteins followed by a sustained release from the caseins [105]. However, in vitro and in vivo evidence suggests that the physical state of the caseins plays an important role in this, i.e., reducing levels of calcium and phosphate associated with the caseins, which compromised casein micelle integrity, leads to weaker gastric coagulation and therefore accelerated gastric emptying [106,107,108]. Changes in the milk matrix as a result of processing have notable effects on aminoacidemia. For instance, heat treatment of milk, which reduces gastric coagulation of milk [109] as a result of whey protein denaturation and casein whey protein interactions, can enhance the rate of post-prandial aminoacidemia [105], whereas inclusion of fat in milk and homogenization can reduce the rate post-prandial aminoacidemia [105] presumably due to increased caloric density as well as the fact that protein-covered emulsion droplets can coagulate in the gastric compartment, leading to creaming [25,105].

Conversion of milk into cheese leads to a matrix that is notably slower in terms of amino acid absorption due to the fact that the casein has been coagulated and concentrated in the cheese-making process [105,110,111]. This matrix is partly broken down due to chewing, but not to a sufficient extent that the proteinaceous material is suitable for gastric emptying [112]. The dense matrix thus requires (extensive) further breakdown of the product matrix, leading to slow gastric emptying and subsequent intestinal digestion and amino acid absorption. Such effects, however, are not directly translatable to all gelled casein matrices because, for yogurt, both enhanced [105] and delayed absorption of amino acids [113,114] have been reported. Such differences are related to the structure of the yogurt, particularly whether it is so-called set-type or stirred-type yogurt. In the case of the former, further breakdown of gelled matrices may be required in the stomach, leading to delayed absorption, whereas for stirred yogurt, particles small enough for gastric emptying are already present in the product, enabling rapid amino acid absorption [105]. From the above examples, it is clear that postprandial aminoacidemia of milk proteins is affected by all aforementioned key determinants of matrices, i.e., the specific components present, the interaction between components and the structural organization.

### 4.2. Dairy and Dental Health

Dental and oral health are an essential part of overall well-being. Dietary factors have been frequently shown to have a large influence on dental and oral health [115,116]. The link between carbohydrates and caries is a frequently highlighted example, with notable differences observed between different carbohydrate sources, with organic acids produced from carbohydrates by the bacteria in dental plaques considered the main cause of caries [117,118,119,120,121]. Of the common carbohydrate sources, lactose has been shown to be the least cariogenic [122,123,124], which may be attributed to the fact that its monosaccharides, galactose and glucose are linked by β-1-4 glycosidic linkage, whereas most other common disaccharides are linked by α-glycosidic linkages, which are far more easily hydrolyzed by micro-organisms [125,126,127,128,129]. Hence, lactose is considered to be poorly fermentable by the oral microbiota and less cariogenic than other carbohydrates. However, when considering dairy consumption and dental and oral health, it is important to look beyond only carbohydrates and focus on complete product matrices. Studies have repeatedly shown an inverse relation between dairy consumption and dental caries [130,131,132,133]. The fact that beneficial effects of dairy consumption on dental health are not only observed for products containing lactose (e.g., milk and yogurt) but also for those containing little or no lactose (e.g., cheese) suggests notable importance of other components of the dairy matrix beyond lactose [134,135,136]. Particularly, protein, calcium, and phosphate have been suggested as important factors in these protective effects through buffering effects as well as by providing essential materials for dental enamel remineralization [134,137,138]. Again, regarding the relationship between dairy and dental health, it is apparent that observed effects cannot be attributed to specific individual components, but are truly matrix effects, which are not easily replicated in, e.g., plant-based drinks [139].

### 4.3. Dairy and Glycemic Responses

As a balanced source of macronutrients, milk is also a source of carbohydrates in the form of lactose. Like all digestible carbohydrates, the consumption of lactose also results in a glycemic response. Lactose is classified as a low-glycemic index (GI) carbohydrate with a reported GI of 46 [140], notably lower than, e.g., other disaccharides such as sucrose (65) and maltose (105) [140,141]. For the monosaccharides, glucose has a GI of 100, whereas fructose and galactose have a reported GI of 23 [142,143]. Purely based on monosaccharide composition, one would thus expect similar GI values for lactose (glucose + galactose) and sucrose (glucose + fructose). For sucrose, the expected GI is perfectly predicted from the GI values of the monosaccharides and accounts for the loss of 18 Da in the formation of the glycosidic bond between the monosaccharides. For lactose, the reported GI value of 46 is almost 40% lower than what would be expected based on monosaccharide composition. This again shows the importance that, even on a disaccharide level, one cannot ‘simply’ consider the monosaccharide constituents but should consider their interaction.

However, when comparing the GI of lactose with that of milk, it is apparent that the GI of milk is even lower, with GI values of milk in the range of 25–48 reported, i.e., 30% lower than what would be expected purely based on lactose, and 50% lower than based simply on monosaccharide composition [143,144,145]. The reason for the lower GI values for milk than for lactose can again be found in the consideration of other constituents present in milk and their interactions and behavior during digestion [143]. Particularly, the gastric phase plays an important role herein, as this phase controls the flow of lactose into the intestine, where it can be hydrolyzed into glucose and galactose, and the resultant monosaccharides are absorbed [140,146]. A more gradual release of monosaccharides into the bloodstream allows for a proportionally higher level of first-pass hepatic clearance of glucose and hence a lower glycemic response [147].

When considering the flow in gastric emptying, there are various factors controlling this process, i.e., control of volumetric flux and caloric flux, but also restriction in particle size [25,148]. When considering milk, there are a number of factors that reduce gastric emptying of the carbohydrate fraction. First, the rather high buffering capacity of milk means that the pH of the stomach content can increase to >6 when an adult consumes a glass of milk on a fasted stomach [149]. Subsequently, substantial amounts of gastric juice are required to reduce stomach pH back to the original pH of ~2; it has been estimated that at this point, milk and gastric juice are at a 1:1 ratio [29], thus reducing effective lactose content in fluid emptied from the stomach. Furthermore, whey proteins have been shown to have a strong effect on glycemic control via stimulating glucagon-like peptide 1 (GLP1) secretion [150,151,152]. GLP1 secretion slows down gastric emptying [153]. Finally, the additional caloric value of fat and protein will further reduce the rate of gastric emptying and thereby modulate glycemic responses [154,155,156]. Overall, it is again clear from the above that when considering glycemic responses after consumption of milk and dairy products, it is not only the fact that lactose is a unique disaccharide but also other non-carbohydrate constituents of milk that play an important role in modulating gastric emptying that results in the observed low glycemic responses. Particularly, the milk proteins and the buffering salts, predominantly phosphate and citrate, are crucial here [157]. This control of gastric emptying is not only crucial for glycemic responses but also for post-prandial amino-acidemia and for absorption of, e.g., Ca, as outlined in subsequent sections.

### 4.4. Dairy and Calcium Bioavailability

When considering the role of dairy products, primarily from bovine milk, in nutrient provision in human diets, minerals are always a key factor, where dairy makes a large contribution. In the Dutch diet, close to 60% of all calcium intake comes from dairy products [158], and comparable contributions of dairy to calcium intake are also seen in many other countries [159]. Key in this calcium intake, however, is not only the fact that dairy products contain high levels of calcium but also that a significant proportion of this calcium is in bioavailable form [160]. Again, the product matrix of dairy products plays an important role in this. Calcium absorption in the small intestine requires for calcium to be in ionic form [161,162]. In the intestinal contents, calcium equilibria will exist between ionic calcium, soluble calcium salts and insoluble calcium salts, the balance of which is governed by concentrations of calcium and calcium sequestering components (e.g., anions and proteins), as well as, e.g., pH and ionic strength [163,164,165]. Furthermore, the absorption rate can be a limiting factor as well, i.e., the presence of (too) high levels of ionic calcium (e.g., from supplements containing soluble calcium salts) in a short time frame will also lead to limited absorption [160,166]. Calcium absorption from dairy products, as well as other food products, is strongly governed by the product matrix [29]. When considering milk as an example, of the approximately 300 mg of Ca that is found in a glass of milk, only one-third is present in the form of soluble salts, whereas the remainder is present in the form of so-called micellar calcium phosphate (MCP) in the casein micelles [163]. These casein micelles are prone to gastric coagulation, thereby delaying their gastric emptying [43,44]. Although pH reduction will lead to the solubilization of MCP from the casein micelles [167,168], this process will be comparatively slow due to the buffering capacity of milk as a result of the presence of phosphates, citrates and proteins [167,168]. Drinking a glass of milk will increase stomach pH > 6 [149], and it will take several hours to reduce the pH back to, e.g., pH~2, during which time the MCP is gradually released [43]. This gradual release of MCP also leads to a gradual emptying of Ca into the intestine and, as a result, gradual absorption [143]. As a result, fractional absorption values of 30–40% of ingested Ca are achieved for intake of typical portions of Ca from milk and dairy products, e.g., 200–300 g per serving [29,169,170]. Fractional absorption can further be increased by dividing intake into more frequent smaller portions, but this, of course, also impacts practicalities in relation to normal consumption patterns [171]. A further important factor of dairy product matrices in relation to absorption of calcium is the absence of anti-nutritional factors, such as phytate and oxalate, which can be present in plant-based sources of calcium [172]. In spinach, for instance, the presence of oxalate results in a very low bioavailability of calcium (<10%) [169,173].

### 4.5. Dairy and Fat Digestion and Absorption

Just like for protein, lactose and minerals, as described in previous sections, the digestion and absorption of dairy fat is also strongly influenced by product matrix effects. As outlined in Section 2, while reductionistic reasonings often linked dairy consumption to increased risks of CVD based solely on SFA content, such findings are typically not supported by RCTs and epidemiological evidence, which can be at least partly attributed to product matrix effects. Control of postprandial lipidemia plays an important role in this. Digestion of fat starts in the stomach, where, if accessible, fatty acids from the sn-3 position of triacylglycerides (TAGs) can be released by human gastric lipase [174,175]. In the intestines, intestinal lipases continue the release of fatty acids from fat. Short-chain fatty acids (SCFA; ≤12 carbon atoms) diffuse directly to the portal vein to be transported to the liver. Long-chain fatty acids (LCFA; >12 carbon atoms) require assembly into mixed micelles to be absorbed and are later, in the intestinal enterocytes, reassembled into TAGs, which become incorporated in lipoprotein particles called chylomicrons [176,177]. Hydrolysis of chylomicrons by lipoprotein lipases can lead to FA uptake by tissues, whereas the remnants of the chylomicron remain in the bloodstream. Their presence has been related to elevated risks of CVD [178,179]. When considering average values for bovine milk fat, ~65% of the fatty acids are classified as saturated fatty acids [180], but ~35% of these are SCFA and would thus not be incorporated in chylomicrons. It should be noted that the fatty acid composition of milk can vary between species [181] and as a result of, e.g., feed [182], which can affect the percentage of fatty acids that would be expected to be incorporated in chylomicrons.

Next to fatty acid composition, the kinetics of fatty acid release and uptake are also important points for consideration. In this respect, gastric emptying and the accessibility of fat to gastric and intestinal lipases play a critical role but also, e.g., the presence of calcium. As outlined before, fat digestion can already occur in the stomach through gastric lipase [174,175]. This, however, is conditional on the TAGs being accessible to the lipase. In raw milk, the milk fat globules are stabilized by the so-called milk fat globule membrane (MFGM), a trilayer structure containing polar lipids, neutral lipids and proteins, which protect the TAG core from the action of gastric lipase [183,184,185]. In commercial milk products, however, the milk has been homogenized, leading to smaller fat globules with a membrane consisting primarily of milk proteins. As a result, the TAG core becomes susceptible to gastric lipase. Homogenization is typically also applied in the production of yogurt but not in cheese because unhomogenized milk is used for the most commonly consumed cheese varieties. For cheese, accessibility of the fat globules for lipase is further hindered by the semi-solid protein matrix in which the fat is embedded and which needs to be digested first before the fat becomes accessible. However, also for milk, the gastric coagulation of the caseins, which can incorporate or entrap fat globules, can hinder lipolysis. This was, e.g., observed in vitro when casein coagulation was modified by varying the casein: whey protein ratio, and higher lipolysis was observed in samples where casein coagulation was weaker [186].

The gastric emptying rate is a key determinant of the entrance of fat into the intestinal environment. It is important to note, in this respect, that milk fat has a notably lower density than water and thus is subject to creaming. The rate of creaming depends on the state of emulsification of the fat. Non-emulsified fat, like in butter, will rapidly cream, whereas the rate of creaming of emulsified fat will depend on the size of the emulsion droplets and/or the aggregates that they are part of. In unhomogenized milk, only some of the largest milk fat globules, which can be up to 10 µm in diameter [30], may cream over the timescale of a few hours in the stomach. However, when milk is homogenized, and all fat globules are typically <1 µm [30], no creaming would be expected in the stomach. However, as outlined before, aggregation of fat globules as a result of enzymatic protein hydrolysis and acid-induced protein hydrolysis can occur, leading to notably larger aggregates that rapidly cream if the fat:protein ratio in the aggregates is sufficiently high to ensure that the density is <1.0 g/mL [187,188,189,190,191]. As a result of these gastric creaming effects, lipidemia may be delayed, as is, e.g., observed in rats, where fat absorption is slower after the consumption of butter than of sour cream [192]. In humans, slower increases in plasma TAGs were also observed for butter compared to Mozzarella cheese or milk [193] and also for non-emulsified vs. emulsified milk fat [194]. This, again, seems in line with higher plasma LDL-cholesterol after a 3-week intervention study for subjects consuming a butter diet rather than a cheese diet [195]. These findings suggest the important role of gastric emptying in controlling postprandial lipidemia and avoiding undesired changes therein.

A further important factor in controlling post-prandial lipidemia is calcium. Soerensen et al. [196] showed that SFA-induced increases in total and low-density lipoprotein (LDL) cholesterol were lower in milk- and cheese-based diets than in a control diet with notably lower calcium content (500 vs. 1700 mg Ca/d). Such effects may be related to the ability of Ca ions to form Ca-soaps of fatty acids with LC-SFA, leading to fecal excretion rather than absorption, as was indeed observed [196]. This is in line with other findings showing decreased post-prandial lipidemia in meals containing higher calcium content [197,198]. Overall, it is thus clear that Ca can notably affect postprandial lipidemia. It is, in this respect, however, important that fat and calcium follow similar gastric emptying patterns.

## 5. Beyond Product Matrix Effects: Meal Effects

While the consideration of product matrices in explaining the physiological impact of intake of nutrients from different products has provided significant advances in the field of human nutrition, this should be considered as a step forward and not as an endpoint. In this respect, it is important to consider that in many cases, products are not consumed individually but as part of a meal containing multiple food products. During consumption and digestion, components from different products can interact or compete for interactions, which can influence the bioavailability of nutrients. Interesting effects, for instance, have been observed in the combined intake of rice and milk, where it was shown that this combination significantly increased the bioavailability of zinc from rice compared to when rice was consumed with water [199]. A positive impact of dairy products on the bioavailability of zinc from cereal products has also been shown, as recently reviewed by Shkembi and Huppertz [200].

The complementarity of food products can also be seen in the area of protein quality. While proteins in some products lack certain indispensable amino acids (IAAs), others contain a surplus and may thus be able to (partially) compensate for deficiencies. This was, e.g., shown for the combination of breakfast cereals and milk [201] or the combination of a bun and a beef burger or pork burger [202]. It is, however, critical that such complementarity is achieved in a single meal rather than over longer timescales, as excess amino acids in the blood will be oxidized [203]. This was illustrated in the feeding of milk replacers to calves, where the synchronous provision of IAAs resulted in a significantly higher nitrogen utilization than the asynchronous supply of IAAs [204]. Next to the complementarity of protein sources, interactions with non-protein constituents of food products within a single meal need to be considered. For instance, the consumption of black tea together with egg protein has been shown to reduce the digestibility and absorption of the latter [205], presumably due to the interaction of tea polyphenols with the proteins. Overall, the above are all examples of a critical yet emerging research domain, wherein food products and food matrix effects should not be considered in isolation but in typical consumption settings because not only the food product itself but also the meal, the bolus and the chyme can be considered matrices.

In addition to specific meal effects, where different products are combined, a further important aspect where matrix effects should be considered is product fortification. Examples include, e.g., the fortification of milk or dairy products with pro- or prebiotics. For probiotics, for instance, the specific product matrix can notably impact both the survival of the bacteria during processing and storage as well as the release of the bacteria from the product matrix in the gastrointestinal tract.

## 6. Conclusions and Future Perspectives

From this review, it is clear that while compositional analysis of food items can provide valuable information on nutrient contents, it cannot be directly linked to nutritional and health impacts. For this, the consideration of food matrix effects is critical because it is not only the presence of nutrients but also their interactions, their structural organization within the product and the resultant physicochemical properties of the product that will determine their bodily function, i.e., the extent to which they can be absorbed and the rate at which this can occur. For dairy products, such matrix effects strongly influence protein and fat digestion and the impact thereof but also influence lactose digestion, the resultant effects of glycemic responses, and even dental health. Further investigation and understanding of matrix and meal effects is critical to support further development and implementation of matrix effects in dietary recommendations.

## Data Availability

No new data were created or analyzed in this study. Data sharing is not applicable to this article.
